# Five-second STEM dislocation tomography for 300 nm thick specimen assisted by deep-learning-based noise filtering

**DOI:** 10.1038/s41598-021-99914-5

**Published:** 2021-10-26

**Authors:** Yifang Zhao, Suguru Koike, Rikuto Nakama, Shiro Ihara, Masatoshi Mitsuhara, Mitsuhiro Murayama, Satoshi Hata, Hikaru Saito

**Affiliations:** 1 Department of Applied Science for Electronics and Materials, Kyushu University, Fukuoka, 816-8580 Japan; 2 Department of Energy Science and Engineering, Kyushu University, Fukuoka, 819-0395 Japan; 3 Institute for Materials Chemistry and Engineering, Kyushu University, Fukuoka, 816-8580 Japan; 4 Department of Advanced Materials Science and Engineering, Kyushu University, Fukuoka, 816-8580 Japan; 5 Department of Materials Science and Engineering, Virginia Tech, Blacksburg, VA 24061 USA; 6 Reactor Materials and Mechanical Design Group, Energy and Environmental Directorate, Pacific Northwest National Laboratory, Richland, WA 99352 USA; 7 The Ultramicroscopy Research Center, Kyushu University, Fukuoka, 819-0395 Japan; 8 Pan-Omics Data-Driven Research Innovation Center, Kyushu University, Fukuoka, 816-8580 Japan

**Keywords:** Characterization and analytical techniques, Imaging techniques, Microscopy, Computer science

## Abstract

Scanning transmission electron microscopy (STEM) is suitable for visualizing the inside of a relatively thick specimen than the conventional transmission electron microscopy, whose resolution is limited by the chromatic aberration of image forming lenses, and thus, the STEM mode has been employed frequently for computed electron tomography based three-dimensional (3D) structural characterization and combined with analytical methods such as annular dark field imaging or spectroscopies. However, the image quality of STEM is severely suffered by noise or artifacts especially when rapid imaging, in the order of millisecond per frame or faster, is pursued. Here we demonstrate a deep-learning-assisted rapid STEM tomography, which visualizes 3D dislocation arrangement only within five-second acquisition of all the tilt-series images even in a 300 nm thick steel specimen. The developed method offers a new platform for various in situ or operando 3D microanalyses in which dealing with relatively thick specimens or covering media like liquid cells are required.

One of the most emerging activities in the field of transmission electron microscopy (TEM) is developing novel techniques for dynamic observation of objects being functioning ideally in their natural environment or artificially controlled environment. Such observation, so-called in situ or operando microscopy, has been advanced by innovative sampling techniques like a liquid cell^1,2^, or functional holders for heating^3–6^ or mechanically deforming^7–12^ a specimen etc. Dedicated “environmental” electron microscopes for controlling atmosphere surrounding a specimen have offered direct observations on the temporal evolution of catalytic systems^13,14^ etc. Ultrafast electron microscopes equipped with a laser-driven electron source have visualized photo-induced material responses^15^ or photon-free electron interactions^16,17^ occurring far beyond the nanosecond time scale. As achieved in some of the above dynamical observation, three-dimensional (3D) TEM such as electron tomography can now provide dynamical analysis of a 3D structure/morphology with a nanometer resolution and in a non-destructive manner, in contrast to other destructive 3D structural analysis methods such as atom probe tomography^18^ and serial-sectioning by focused ion beam – scanning electron microscopy (FIB-SEM)^19,20^. Super-resolution microscopy^21^ is a non-destructive method enabling a nanometer resolution, however, its application is limited to objects where fluorescent probes can be attached.

While 3D atomic structure analysis has shown remarkable results for isolated single nanoparticles^22–26^, the low penetrability due to the strong interaction of electron beam with matters often problematic in 3D TEM analysis. This becomes severe when the targeted objects are nanometer sized and embedded in other materials as well as liquid, or are part of a larger-scale object. Generally speaking, the thickness of a specimen for TEM analysis is required to be thinner than 100 nm, and this limitation becomes strict in the conventional TEM (CTEM) mode due to image blur caused by inelastic scattering, which cannot be overcome without adding dedicated equipment like a recently developed chromatic aberration corrector^27^.

On the other hand, in the scanning TEM (STEM) mode which is basically free from the chromatic aberration of the imaging lens system, the deterioration of image quality due to the specimen thickness is much smaller than that in the CTEM mode. In fact, a 3D arrangement of dislocations in an iron slab, a 400 nm thick specimen, was clearly visualized by STEM tomography^28^. In this particular study using a 300 keV electron beam, dislocation line contrast was visible even when the specimen was tilted by 60°, i.e., the effective specimen thickness reached to 800 nm. It was also demonstrated that STEM tomography overwhelmingly effective for a very thick biological specimen^29^, where even a 1-µm-thick specimen was successfully analyzed in three dimensions. As these previous examples demonstrate, the STEM mode has a great advantage over the CTEM mode for the 3D visualization of nanostructures inside thick specimens. Furthermore, a high-angle annular dark-field imaging method is available for the STEM mode, which is suitable to quantify the mass density and chemical composition from the image intensity^30^.

Operando observation also requires its temporal resolution sufficient to observe the time evolution of the targeted phenomenon. In principle, the STEM mode, which is a serial detection method, has disadvantages in terms of imaging speed compared to the parallel detection method counterparts such as the CTEM mode. Recently developed high-speed cameras for the CTEM mode have achieved a frame time of sub-millisecond order, enabling the CTEM mode to implement dynamic structural analyses down to molecular scale^31,32^. When a high-speed camera is applied to fast CTEM tomography, a few second of acquisition time for tilt-series images is archived^14,33^. In this respect, the acquisition time for STEM tomography is still in the order of minutes^4–6,34^ due to the lack of a fast-imaging method enabling millisecond order frame time for the STEM mode.

In order to pave a new way to operando 3D observation and make structural analysis available for thick specimens, here we propose a novel approach based on a deep learning method to solve the problem inherent in fast imaging in the STEM mode, i.e., non-trivial noises superimposed in images, which is nearly impossible to remove by conventional noise filtering methods. We applied our new method to STEM imaging of dislocations in a steel specimen having 300 nm thickness, and successfully demonstrated that the quality of images taken with 30 ms per frame was significantly improved by our unique noise filtering method based on the U-Net^35^. The result images are almost equal to the quality of 50 frames averaged images composed by the drift-corrected frame integration (DCFI) technique^36^, exhibiting sufficiently high signal-to-noise ratio for 3D structure analysis. Then, applying this U-Net-assisted STEM imaging technique to the rapid acquisition of tilt-series images for tomography that accomplishes five seconds of acquisition time for tilt-series images. In comparison to the conventional method requiring few tens of minutes, our 5-s STEM tomography represents the 3D dislocation arrangement accurately enough to understand its structure in mesoscopic scale. The present challenge also highlights an unprecedented aspect of deep-learning-based noise filtering step that overcomes complex degradation of image quality caused by a rapid scan, showing promise for the application of rapid STEM imaging approach to various in situ or operando experiments.

## Deep-leaning-assisted rapid STEM tomography

### Concept of the proposed method

**Figure 1:**
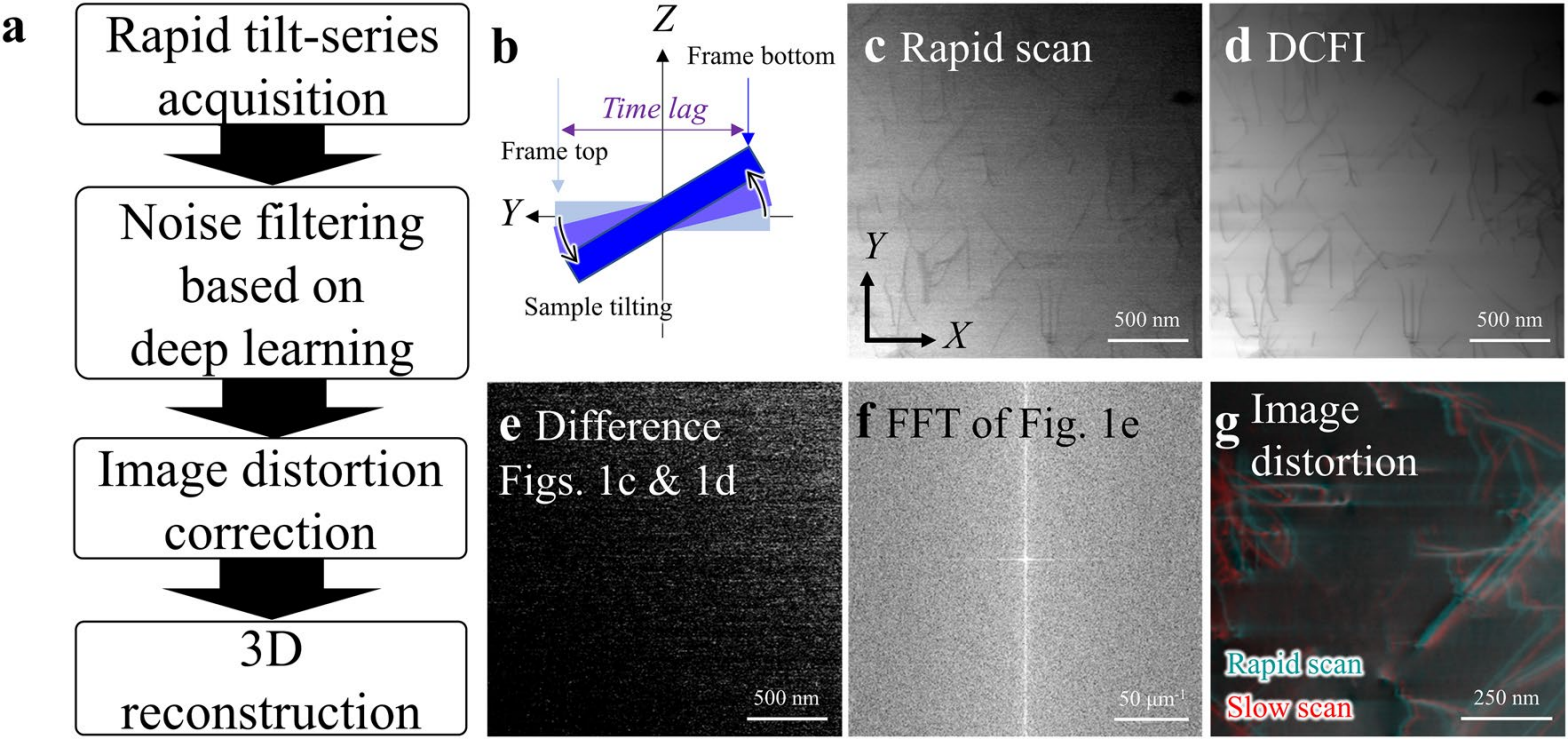
Problems to be solved and outline of the proposed protocol for rapid STEM. (**a**) Procedure for the proposed rapid STEM tomography. (**b**) Schematic drawing of time lag artifact. (**c**) Typical image of dislocations obtained with a single rapid scan. The scan speed and frame time are 114 ns/pixel and 30 ms, respectively. (**d**) Averaged image by 50 equivalent rapid scan images for the field of view corresponding to (**c**). (**e**) Typical noise image produced by subtracting the averaged image (**d**) from the single rapid scan image (**c**). (**f**) Fast-Fourier-transformed (FFT) image of (**e**). (**g**) Superposition of a rapid scan image (aqua, 114 ns/pixel) and a corresponding slow scan image (red, 6.1 µs/pixel) for the same field of view. Note that the scale bars attached with the images include 10% errors at most because all the STEM images here are not processed with the distortion correction.

We have developed a rapid STEM tomography method composed of three parts: (1) rapid tilt-series image acquisition (less than five seconds), (2) image noise filtering and distortion correction, and (3) three-dimensional (3D) reconstruction (Fig. 1a). The rapid tilt-series image acquisition was conducted under 114 ns/pixel of scan speed and 28 degree/s of specimen specimen-tilt speed. These scan and specimen-tilt speeds are the fastest settings available in the electron microscope used in this experiment. This fastest scan speed achieves about 30 ms frame time, the time it takes to render a 512 × 512 pixels image size, which is hundreds of times faster than the conventional frame time for STEM imaging, although the resultant low quality of original images (Fig. 1c) needs to be compensated by a noise filtering technique. This extremely fast scan speed is required to reduce the time lag artifact negatively impacting the accuracy of the final 3D reconstruction. The time lag artifact occurs when the specimen tilt advances during a frame acquisition due to the continuous tilting adding a curl shape distortion to the final 3D reconstruction (Fig. 1b). Even within the 30 ms of image acquisition time, the specimen swings by 0.8° in the current experimental setting (28 degree/s), resulting in a tiny time lag artifact as discussed later. Note that, as another method for tomography than tilt-series image acquisition, we can consider through-focal image acquisition^37^ which is completely free from any artifacts or limits accompanied with specimen tilting. This technique can also be effectively combined by noise filtering techniques discussed in this paper, and is a potential strategy for further speeding up of operando 3D observation although it is beyond the scope of this paper.

The extremely fast scan causes a severely low signal-to-noise ratio, as shown in Fig. 1c. To resolve the problems associated with the severely low signal-to-noise ratio, a deep-learning-based noise filtering method was developed in this study. Noise filtering algorithms for digital images are categorized into two types, i.e., algorithms with and without learning processes. The block-matching-and-three-dimensional filtering (BM3D) algorithm is a well-known powerful denoising technique not requiring any prior learning processes^38,39^. In general, non-learning noise filtering algorithms mainly estimate the variance of an additive white Gaussian noise (AWGN). The latest BM3D algorithm provided by Egiazarian et al. has been developed to optimize the filtering process by foresight knowledge. This algorithm works effectively when the existing noise matches with one of pre-registered models like band-pass noise and line pattern noise, etc., thus, the type and amplitude distribution of potentially existing noises in each image have to be known or well estimated in advance^39^. However, identifying the noise model becomes too complex when an image includes non-trivial noises originating from the characteristics of the devices used for the imaging, which become significant or non-negligible in extremely fast scan settings pushing the beam scan device and /or the electron detector to the limit. In fact, this kind of non-trivial noise was recognized in our experiment, as shown in Figs. 1c–f. Some systematic patterns appeared in Fig. 1e by subtracting an averaged image (Fig. 1d) from a single rapid scan image (Fig. 1c), which seemed to be streaked in the fast scan direction (*x* axis) and had higher frequency components in the *y* direction as shown in Fig. 1f. These observations led us to develop a deep-learning-based noise filtering method, being inspired by previous successful applications for some reconstruction problems from noisy or incomplete inputs^40–44^.

It should be noted that the rapid scan also introduced enormous image distortion, as shown in Fig. 1g, probably due to the hysteresis of the beam scan device consisting of a set of magnetic coils. In Fig. 1g, two images were taken from the same field view at two different scan speed settings were superimposed and compared. In our electron microscope, image shrinkage in the *x* direction appeared when the scan speed reached 114 ns/pixel. This image distortion cannot be expressed by affine transformation, i.e., it is not a uniform shrinkage of the whole image. In order to calibrate the dimensions of the rapid scan images, we measured this position-dependent distortion by using a commercial standard specimen, a cross grating pattern made of gold nanoparticles. The technical details of this image distortion calibration are described in the method section and Supplementary information A.

All the artifacts we have discussed so far appear specifically in the STEM mode while they have not been seen in the CTEM mode, and this fact is likely to be a reason why tilt-series image acquisition with a few seconds order has only been implemented in the CTEM mode combined with a high-speed camera. However, those shortcomings in the STEM mode can drastically be improved just by software developments as discussed in this paper.

### U-Net-based noise filtering

**Figure 2:**
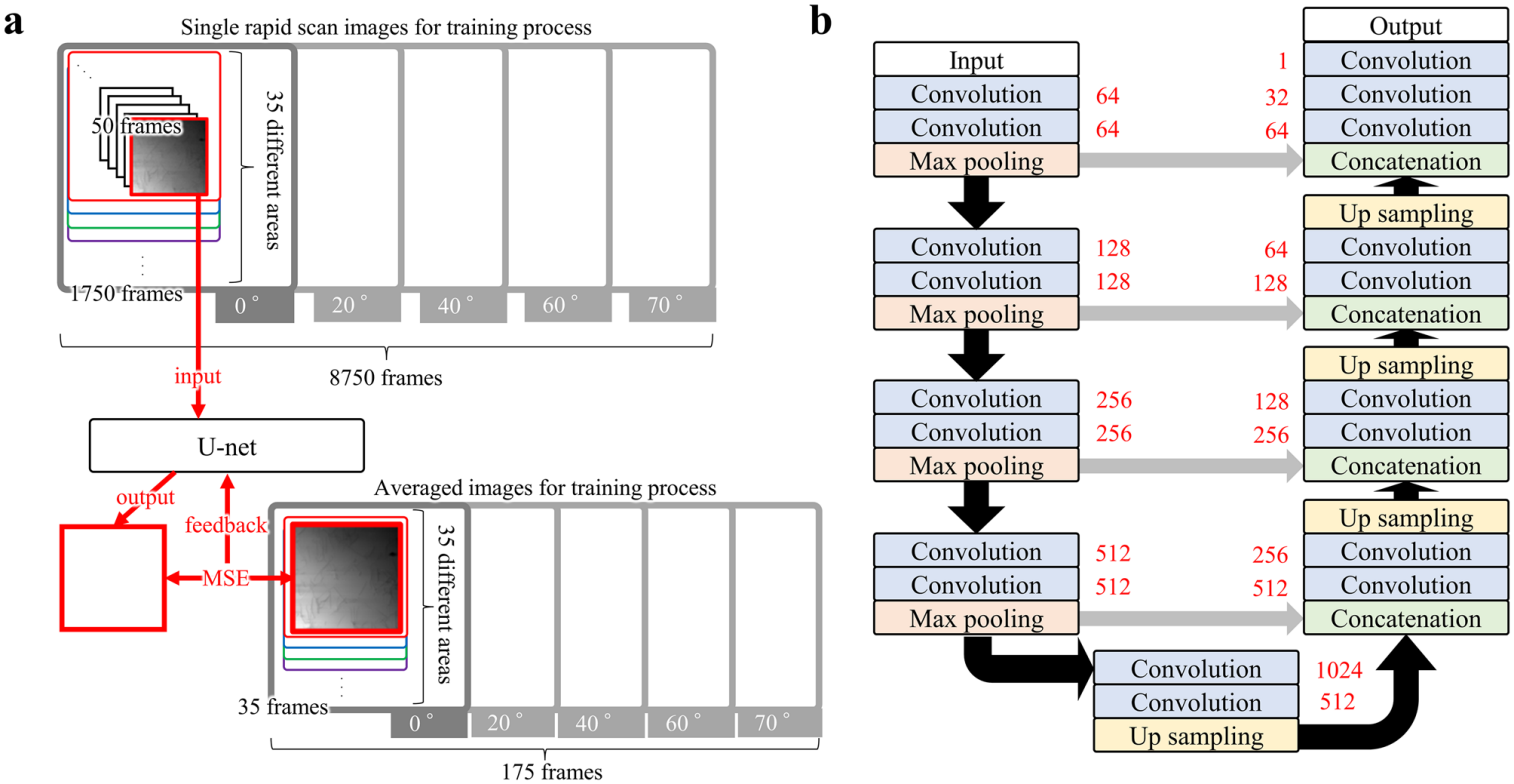
Deep learning by single rapid scan images and averaged images. (**a**) Schematic drawing of deep learning employed in this study. (**b**) Architecture of U-Net. Each number listed on the side of the boxes indicates the number of feature maps. The gray arrow indicates the concatenation process.

Supervised learning was employed for developing a dedicated noise filter finely adjusted to the noise specific to the hardware and the operating condition used in this study. The training data used for machine learning consists of 175 different areas in total, taken from five different specimen tilt angles (35 areas per angle) under the same imaging condition as the rapid tilt-series images regarding the scan speed, the pixel size, the electron optics, the detector setting, etc. 50 frames (images) were acquired from each of these areas; in summary, 8750 rapid scan images were the inputs for to be developed U-Net’s training as described in Fig. 2a. These noisy images were used intentionally because these would have a similar image quality with unprocessed tilt-series images expected from this rapid STEM tomography method. We also prepared one reference image for each of the areas by averaging the 50 frames (images) using the drift-corrected frame integration (DCFI) function equipped in Titan’s Velox™ software. The U-Net was optimized through the training process so that each of the output images became similar to the corresponding reference image (Fig. 2a). This training process possibly becomes more efficient by employing recent algorithms such as Noise2Noise^45^ and Noise2Void^46^ so that the time cost for data collection is reduced.

The effective thickness, the pathlength of the electron beam has to penetrate through, depends on the tilt angle of the specimen slab and causing the tilt-angle-dependent signal-to-noise ratio. This characteristic suggests that the training data should include various images from different tilt angles ranging from 0° to 70°. Therefore, multiple areas and specimen tilt angles were chosen for collecting the training data. In order to ensure that the areas for the training data have a similar thickness as the area used for the rapid STEM tomography, we only selected the areas where the image intensity histogram is similar to the corresponding data for the rapid STEM tomography as discussed in Supplementary information B. In this way, we collected 35 averaged images and 1750 rapid scan images at 5 different specimen tilt angles 0°, 20°, 40°, 60° and 70°, respectively (Fig. 2a).

We used Dragonfly™ software (Object Research Systems) for the U-Net-based noise filtering. The used architecture is described in Fig. 2b, which is a standard one known as U-Net^35^. Prior to input into the U-Net, the brightness of all the experimental images as well as the reference images was adjusted the way that the minimum intensity in each of the images equaled to zero, and all the images were then normalized by the maximum intensity in each of the images. We set the patch size to 64 × 64 pixels, the optimization algorithm to Adam^47^, and the objective function to mean square error.

## Results and discussion

### Comparison of U-Net and BM3D

**Figure 3:**
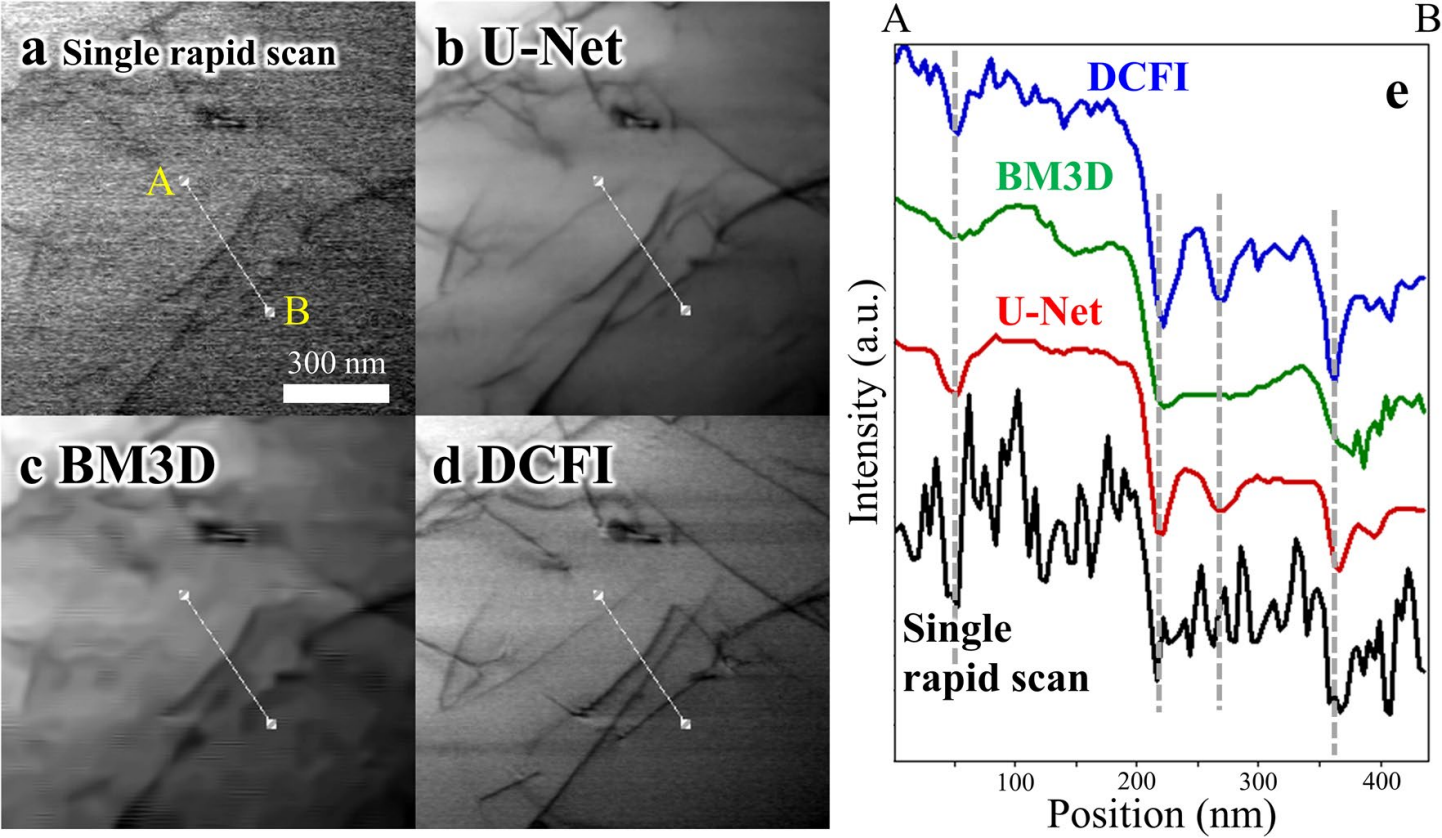
Performance of the U-Net-based noise filter. (**a**)–(**d**) Comparison of (**a)** single rapid scan image, (**b**) filtered image by the U-Net, (**c**) filtered image by the BM3D, and (**d**) DCFI image using 50 equivalent rapid scan images for the same field of view. (**e**) Line profiles extracted from the four images (Figs. 3a-3d) along the indicated lines (from A to B).

In the U-Net-based noise filter development process, we have evaluated the performance of the U-Net-based noise filter by applying it to multiple new fields of views not included in the training data. Figure 3 shows a comparison between an original single rapid scan image and two filtered images, where dislocations in an austenitic steel are visualized with dark lines. The original bright-field STEM image was obtained under excitation of an *hkl* = 200 diffracted beam (details of the observation condition are described in the method section and Supplementary information C). The image processed through the U-Net-based noise filter (Fig. 3b) appears much clearer than the original image (Fig. 3a). One can intuitively recognize that the quality of the noise-filtered image is closer to that of the DCFI image (Fig. 3d). This is quantitatively confirmed by the evaluation based on the peak signal-to-noise ratio (PSNR), summarized in Table 1, where the PSNR was calculated relative to the averaged (DCFI) image. Thus, each PSNR value indicates how the image is similar to the DCFI image. The PSNR values, the mean and standard deviation, were calculated for 10 new fields of views selected from images taken at the specimen tilt angle 0°, 20°, 40°, 60° and 70°, respectively (see typical test images taken at different tilt angles in Supplementary information D).

**Table 1:** Peak signal-to-noise ratio of noise-filtered images by U-Net-based algorithm and BM3D-based algorithm.

	Method	
	U-Net (dB)	BM3D (dB)
**Tilt angle**		
0°	30 ± 2	34 ± 3
20°	29 ± 3	32 ± 2
40°	32 ± 2	33 ± 2
60°	32 ± 2	35 ± 3
70°	30 ± 5	35 ± 3

The performance of the U-Net-based noise filter is compared with the BM3D-based filter which was optimized for this study (Supplementary information E). As shown in Table 1, the BM3D-based noise filter gives higher scores regarding the PSNR. However, the line-shaped strain contrast arising from dislocations became remarkably broadened in the width direction in the image processed through the BM3D-based noise filter (Fig. 3c). This is more clearly shown in the contrast profiles extracted from the images (Fig. 3e). The differences between the U-Net-based and BM3D-based noise filters are summarized in Tables 2 and 3 regarding the width and intensity of the dark lines relative to those in the DCFI images, respectively.

**Table 2:** Relative width of dislocation dark line visualized in the noise-filtered images by U-Net-based algorithm and BM3D-based algorithm.

	Method	
	U-Net (%)	BM3D (%)
**Tilt angle**		
0°	98 ± 19	125 ± 27
20°	95 ± 26	142 ± 58
40°	110 ± 15	159 ± 42
60°	110 ± 23	175 ± 54
70°	123 ± 31	173 ± 46

**Table 3:** Relative intensity of dislocationdark line visualized in the noise-filtered images by U-Net-based algorithm and BM3D-based algorithm.

	Method	
	U-Net (%)	BM3D (%)
**Tilt angle**		
0°	93 ± 11	94 ± 19
20°	81 ± 33	76 ± 29
40°	95 ± 11	85 ± 19
60°	79 ± 16	71 ± 9
70°	95 ± 17	83 ± 18

The details for measurement of the relative width and intensity are described in Supplementary information D. A large difference is found in the relative width, whereas the relative intensity is comparable. The line broadening becomes larger with increasing the tilt angle in both filters. The reason for this tilt angle dependence is considered to be the effective specimen thickness increasing with the tilt angle; that is, the signal-to-noise ratio is deteriorated in the higher tilt angle range due to a decrease of forward-scattered electrons. That said, the U-Net-based noise filter keeps the line broadening smaller than the BM3D-based filter. Even when the tilt angle reached higher than 60°, i.e., the effective thickness became thicker than twice the original thickness, the line broadening was suppressed to be less than 150%, while it sometimes became more than 200% with the BM3D-based noise filter. Accordingly, although the U-Net-based noise filter gives a slightly lower score of PSNR than the BM3D-based one, our U-Net-based noise filter is obviously superior to the BM3D-based one by setting the highest priority on the performance on spatial resolution, especially in the application to rapid STEM tomography. It could be worth noting that so much difference in performance between the two noise filters was not recognized in the previous applications for noisy images obtained in the CTEM mode^47^. The contrasting results seen in this study suggest that the noise contained in the rapid scan images is not representative, and thus, fine tuning of the noise filter based on supervised learning is especially required in the rapid STEM imaging.

### Evaluation of the U-Net-assisted rapid STEM tomography

**Figure 4:**
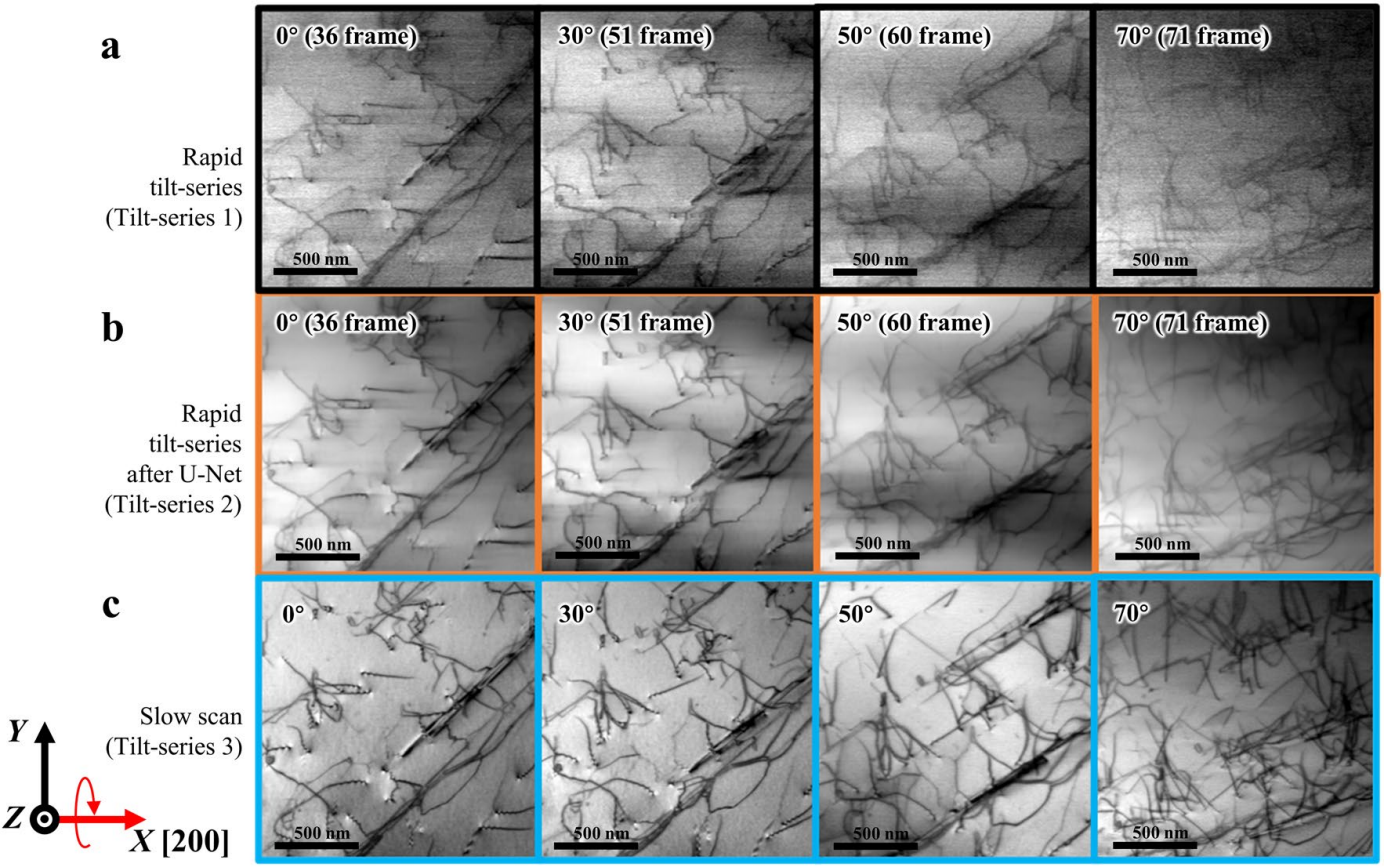
Application of the U-Net-based noise filter for rapid tilt-series images. (**a**) Part of original rapid tilt series (Tilt-series 1). (**b**) Denoised rapid tilt series generated from Tilt-series 1 (**a**) through the U-Net-based filter (Tilt-series 2). (**c**) Corresponding slow tilt series obtained by a conventional method (Tilt-series 3). The *X*, *Y*, and *Z* axes depicted at the bottom left represent coordinates fixed at the used TEM instrument.

Here we show the initial result of rapid STEM tomography assisted by the U-Net-based noise filter and discuss its performance by comparing it to a dataset obtained from a conventional method, i.e., using an intermittent specimen tilt and slow scan speed (see the method section for the detailed acquisition parameters). The fastest scan speed, 114 ns/pixel, was used to acquire a single STEM image of 512 × 512 image size (frame time 30 ms), although the actual imaging speed became 14 fps because an additional 40 ms to store a frame before scanning the next frame was required. This imaging speed provides tilt-series images taken every 2° on average for the fastest specimen-tilt speed of 28 degree/s. Under this experimental condition, 71 frames were collected over a 140° of angle range during 5 s of total acquisition time as a single set of tilt-series images (Tilt-series 1). Several selected images are shown in Fig. 4a, which are compared with denoised images processed by the U-Net-based noise filter (Tilt-series 2, Fig. 4b) and tilt-series images obtained by a conventional method with a slow scan speed (Tilt-series 3, Fig. 4c). All the images in Fig. 4 were processed by a distortion correction (see the method section and Supplementary information A).

**Figure 5:**
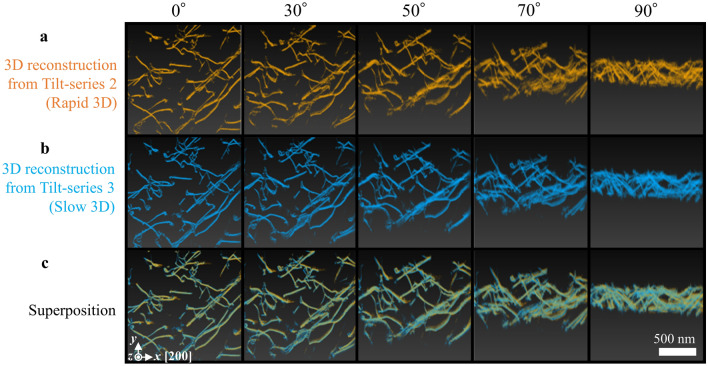
Rapid STEM dislocation tomography. (**a**) and **(b**) 3D-reconstructed data calculated from (**a**) Tilt-series 2 and (**b**) Tilt-series 3. (**c**) Superposition of Figs. 5a and 5b. The 3D data are projected along with several angles and displayed as 2D images. The *x*, *y*, and *z* axes depicted at the bottom left represent coordinates fixed at the used TEM instrument.

**Figure 6:**
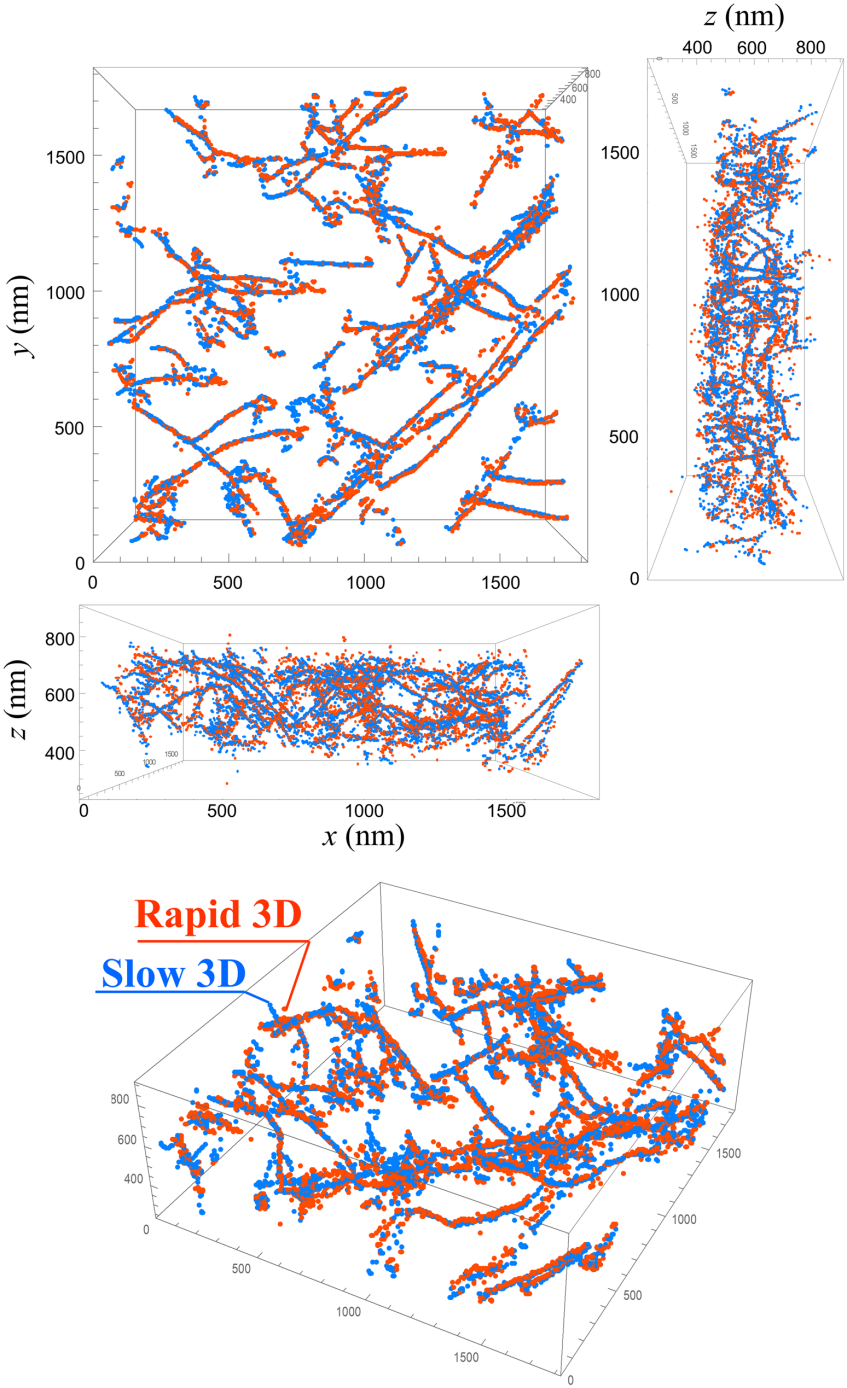
3D plot of local intensity centers. Orange and aqua plots were extracted from Rapid 3D (Fig. 5a) and Slow 3D (Fig. 5b), respectively.

Figure 5a–c shows results of 3D reconstruction from the Tilt-series 2 (noise filtered Rapid 3D) and Tilt-series 3 (Slow 3D) and their superposition, respectively. The three-dimensional spatial arrangement of dislocations in both Rapid 3D and Slow 3D is almost the same, suggesting that the U-Net-based noise filter effectively discriminated the signals from severe noises in the Tilt-series 1. In order to precisely evaluate the accuracy of 3D positional measurement, the center of each of the dislocations was extracted from both Rapid 3D and Slow 3D. Here the center of *n*th dislocations in the *k*th *yz* cross-section is defined as a weighted average of two-dimensional (2D) positions ${\mathbf{r}}_{nk}$;

1 $${\mathbf{G}}_{nk}=\left(\begin{array}{c}{G}_{y,nk}\\ {G}_{z,nk}\end{array}\right)=\frac{\sum I\left({\mathbf{r}}_{nk}\right){\mathbf{r}}_{nk}}{\sum I\left({\mathbf{r}}_{nk}\right)},$$

where $I\left({\mathbf{r}}_{nk}\right)$ is the intensity at the position ${\mathbf{r}}_{nk}$, and the summation was performed near the dislocation of interest (see Supplementary information F). This definition based on the weighted average is more robust against noise than the definition based on the local maximum. ${\mathbf{G}}_{nk}$ for both Rapid 3D and Slow 3D are plotted in Fig. 6. The nearest points across Rapid 3D and Slow 3D were searched in the same *k* of cross-sections to determine the pairs, i.e., ${\mathbf{G}}_{nk,rapid}$ and ${\mathbf{G}}_{nk,slow}$. The average of positional errors in the *y* and *z* directions is defined as follows;

2 $$\overline{\left|\Delta {G}_{y}\right|}=\frac{1}{N}\sum \left|{G}_{y,nk,rapid}-{G}_{y,nk,slow}\right|,$$

3 $$\overline{\left|\Delta {G}_{z}\right|}=\frac{1}{N}\sum \left|{G}_{z,nk,rapid}-{G}_{z,nk,slow}\right|,$$

where *N* is the number of found pairs in the entire space. $\overline{\left|\Delta {G}_{y}\right|}$ and $\overline{\left|\Delta {G}_{z}\right|}$ are 7.8 nm and 10.3 nm, respectively. It should be noticed that there are some missing parts of the dislocations in Rapid 3D probably due to the weaker contrast than the detection limit, although they are a very small fraction of all the dislocations in the field of view. However, such a difference in detection is not what we evaluated here. To purely evaluate the positional accuracy, only the corresponding points detected in both Rapid 3D and Slow 3D were selected in the above calculations of $\overline{\left|\Delta {G}_{y}\right|}$ and $\overline{\left|\Delta {G}_{z}\right|}$. In the same way, we extracted the local intensity centers in the *xz* cross-section. The average positional error in the *x* direction $\overline{\left|\Delta {G}_{x}\right|}$ was calculated to be 6.4 nm. The calculated positional errors in the *x* and *y* directions are less than 0.5% of the lateral sizes of the field of view. In particular, the error in the *x* direction ($\overline{\left|\Delta {G}_{x}\right|}$) is the smallest, indicating that the influence of nonlinear image distortion due to the rapid scan is successfully removed by the correction method discussed in Supplementary information A. On the other hand, $\overline{\left|\Delta {G}_{z}\right|}$ is slightly larger than the other two directions, suggesting anisotropic errors degrading the positional accuracy in the *z* direction.

**Figure 7:**
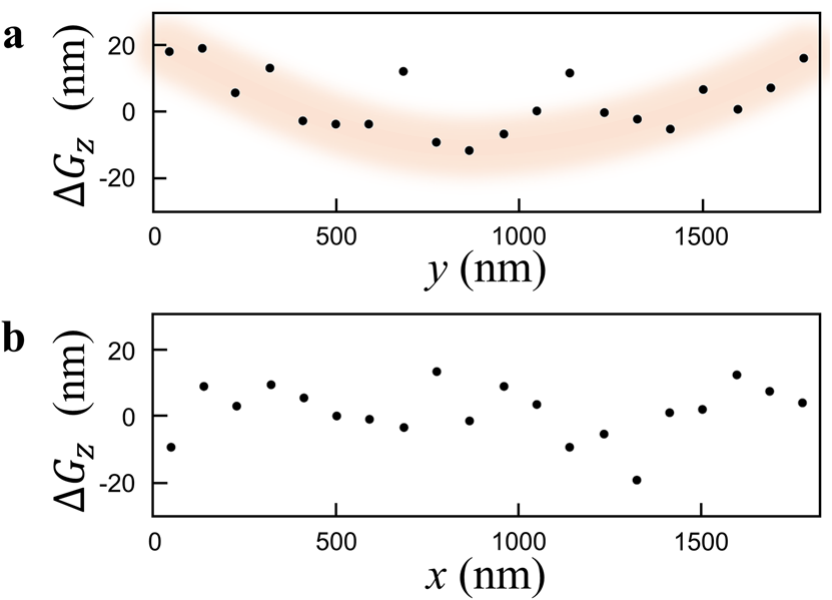
$\Delta {G}_{z}$ calculated from the 3D plot of local intensity centers. **(a**) *y* dependence of $\Delta {G}_{z}$ averaged along the *x* direction. (**b**) *x* dependence of $\Delta {G}_{z}$ averaged along the *y* direction.

In order to further investigate the morphological difference between Rapid 3D and Slow 3D, $\Delta {G}_{z}$ is plotted as a function of *y* and as a function of *x*, where they are averaged regarding the *x* and *y* directions, respectively (Fig. 7a and b). A characteristic convex trend appeared in the *y* dependence of $\Delta {G}_{z}$ (Fig. 7a) while no obvious trend was recognized in the *x* direction (Fig. 7b), indicating that the specimen looked as if it curled up around the tilt axis (*x* axis) during acquiring of the Tilt-series 1. This convex dependence of $\Delta {G}_{z}$ in the *y* direction is the time lag artifact inevitably appearing in STEM tomography with a continuous specimen tilt (Fig. 1b). The time lag becomes the largest at the specimen tilt angle equal to 0° because the field of view becomes the largest in the *y* direction. At that time, the *z* component is dominant in the specimen movement. This is the reason why the time lag artifact mostly affects the accuracy of *z* positions. Note that this artifact is reproducible in principle, and thus, the positional accuracy in the *z* direction can be further improved by a proper calibration. Overall, we can conclude that the measured position of a dislocation in 3D by the proposed rapid STEM tomography is accurate enough to characterize the mesoscopic scale slip systems of materials.

## Conclusions

To conclude, we have herein demonstrated a rapid STEM tomography, which drastically shortens the acquisition time down to 5 s, approaching to TEM-based rapid tomography. This method potentially improves the temporal resolution of operando 3D observation. The U-Net-based noise filter nearly completely recovers image quality from the poor images severely suffered from low signal-to-noise ratios, enabling a short frame time (30 ms) STEM imaging. This filter exhibits higher performance than the widely used BM3D filter in terms of keeping the original contour with a high resolution. It has been demonstrated that the deep-learning-based noise filtering plays a critical role for pushing the temporal resolution of STEM imaging up to a hardware limit. This promising approach currently has a large room to be improved especially in terms of time cost reduction or simplifying the collection procedure of training data. This point should be kept considering through comparison with various other learning algorisms as a future task, and it should be examined for various types of applications. The nonlinear image distortion caused by the rapid scan can be compensated by the correction algorithm using a standard calibration specimen. As a result, 3D dislocation arrangement in an austenitic steel was clearly visualized, which well reproduced the result obtained by the conventional method based on intermittent specimen tilt with the slow scan of 1.6 s per frame. The averaged positional errors of the rapid STEM tomography were less than 8 nm in the lateral directions relative to the 3D data obtained by the conventional method, which were less than 0.5% of the field of view (~ 1.8 µm). The positional error in the *z* direction was relatively large (10.3 nm) due to the time lag artifact, which is expected to be further reduced by developing an appropriate calibration process. In addition, such an artifact due to the current limitation of scan speed is to be basically and continuously reduced, being expected by a hardware development going on^48^. It is significant to materials characterization especially for nano-mechanics that the present method enables tomographic observation of a 300 nm thick steel specimen which is practically impossible by the conventional TEM tomography. Several significant technical developments in STEM-based postmortem analysis techniques have been made over last several years aiming to high-spatial resolution structural defects characterization. These aim to obtain quantitative information from a thicker specimen (foil thickness > 200 nm) than conventional TEM approach by removing undesired diffraction contract elements such as bend contour^49^ or by distinguishing dislocations having different crystallographic characters at the same time based on their appearance^50,51^. In comparison with conventional TEM based approach, these techniques appear to be more efficient to identify the crystallographic nature of dislocation loops down to about 2 nm in diameter and to provide statistically relevant number density of dislocation loops and other irradiation and/or plastic deformation induced defects. Accordingly, the proposed technique would greatly contribute to recent research activities as a platform technique for the four-dimensional (4D) observation requiring thick specimens such as dislocations in plastic deformation^52–54^.

## Methods

### Specimen preparation

A single crystal of an AISI316L alloy, a typical austenitic stainless steel, was employed for a specimen. The chemical composition of the alloy was Fe-0.012% C-0.45% Si-0.17% Mn-17.3% Cr-10.57% Ni-4.72% Mo (mass %). The main phase of the as-prepared alloy is austenite (*γ*-Fe), whose crystal structure is the face-centered cubic (fcc) structure. After solid-solution heat treatment at 1323 K and 2 h, dislocations were introduced by a compressive deformation at room temperature. The compressive direction was nearly parallel to the < 100 > _*γ*-Fe_ direction. The strain rate during the deformation was 1 × 10^−4^ s^−1^ and the magnitude of compressive plastic strain was about 3%. The specimen was cut into square sheets of 1.5 mm × 1.5 mm × 0.05 mm in size and then electropolished in an HClO_4_–CH_3_OH electrolyte under conditions of 20 V, 20 mA and 243 K.

### Instrumentation

A transmission electron microscope Titan Cubed G2 (Thermo Fisher Scientific Inc.) was operated at an acceleration voltage of 300 kV under the STEM mode with a relatively small convergence semi-angle of the incident electron beam, 1.2 mrad. This small convergence angle makes the depth of focus sufficiently deeper so that the influence of the inevitable defocus caused by the high-angle tilt of the specimen can be ignored. In this setup, the blur due to such a defocus is less than 2 nm at most. All the images in this study were acquired using the commercial software, Velox™ (Thermo Fisher Scientific Inc.). The pixel size of all the images was 4.56 nm. A high-angle triple-axis (HATA) specimen holder (Mel-Build Co.) was used for adjusting the crystal orientation in order to maintain the two-beam excitation condition over the whole range of specimen tilt angle, which is required to visualize the same set of dislocations in all the tilt-series images^55,56^. In this experiment, the [200] direction of the specimen was aligned to the tilt axis of the specimen holder (Supplementary Information C). All the dislocation images were bright-field images obtained by detecting the direct beam disk.

### Rapid tilt-series acquisition

For tilting the specimen, a goniometer of the microscope was rotated from -70° to + 70° at the fastest speed which the regular user mode can choose. The resultant duration from the start to stop rotation was about 5 s, which was the shortest value achievable within the hardware limit on our microscope. For the selected image size, 512 × 512 pixels, it takes about 30 ms to read a frame at a scanning speed of 114 ns/pixel and about 40 ms to store the frame, which means that it takes about 70 ms in total to completely record one frame. During the 5 s, about 70 frames could be acquired without stopping the goniometer rotation throughout the tilt angle range, from − 70° to + 70°, which is not inferior to the number of tilt-series images acquired by a conventional method. In order to evaluate the quality of rapid STEM tomography comparing to the conventional method, we also acquired tilt-series images from the same field of view in the almost same condition but taking a longer frame time of 1.6 s and intermittent manipulation of the goniometer as performed in the previous studies^28,56^. The tilt-series images were acquired every 2° throughout the range from − 70° to + 70°. In this conventional method, the rotation of the goniometer was stopped during image acquisition of 1.6 s at each tilt angle, resulting in dozens of minutes for the total procedure of tilt series image acquisition.

### Image distortion correction

As shown in Fig. 1g, the rapid scan image taken at 30 ms/frame is shrunken only in the *x* direction, compared with the slow scan taken at the 3 s/frame. This image distortion direction matches with the fast scan direction (Fig. 1g), suggesting that this image distortion is related to the characteristic of the beam scan device. In this study, we assume that nonlinear image distortion can be ignored in the slow scan. Under this assumption, we derive the image distortion distribution in the rapid scan image by calculating the local cross-correlation between the rapid scan image and the slow scan image obtained from the identical field of view as discussed in Supplementary information A.

### Image processing and 3D reconstruction

The effective thickness changes due to the specimen tilt cause the dislocation contrast being inconsistent. The tilt-series images we collected were not strictly a projection of the dislocation structure, so they cannot be directly applied to 3D reconstruction. Therefore, we implemented a binarization processing to the tilt-series images prior to 3D reconstruction (see the details of the binarization in Supplementary information G).

3D reconstruction also requires the tilt angle information of the tilt-series images. However, due to the non-uniform motion of the rapid specimen tilt, the tilt angle of each frame of the rapid tilt-series images cannot be accurately known solely from the averaged rotation speed of the goniometer. To resolve this problem, we use multiple feature points in each of the images to correct tilt angles of each frame as discussed in Supplementary information H.

After the above steps, we used Inspect3D™ software (Thermo Fisher Scientific Inc.) to align the tilt-series images and reconstructed the 3D datasets by the simultaneous iterative reconstruction technique (SIRT) with 50 times iterations. All the tilt-series images of rapid and conventional methods were used for 3D reconstruction, and Visualizer-evo™ software (SYSTEM IN FRONTIER INC.) was then used for 3D display.

## Supplementary Information

Supplementary Information 1.

Supplementary Video 1.

Supplementary Video 2.

Supplementary Video 3.

Supplementary Video 4.

Supplementary Video 5.

Supplementary Video 6.

Supplementary Video 7.

Supplementary Video 8.

## Data Availability

The codes employed for the noise filtering and distortion correction are available from the corresponding author upon reasonable request.
