# Supplementary material for: Five-second STEM dislocation tomography for 300 nm thick specimen assisted by deep-learning-based noise filtering
Source: Sci Rep. 2021 Oct 26;11:20720. doi: 10.1038/s41598-021-99914-5 (PMC8548491; doi:10.1038/s41598-021-99914-5)
Supplement: Supplementary file 1 — Supplementary Information 1. [file 41598_2021_99914_MOESM1_ESM.pdf]

Supplementary Information

Five-second STEM dislocation tomography for 300 nm thick specimen assisted by deep-learning-based noise filtering

Yifang Zhao[1], *Suguru Koike*[1], *Rikuto Nakama*[2], *Shiro Ihara*[3], *Masatoshi Mitsuhara*[4], *Mitsuhiro Murayama*[3,5,6], *Satoshi Hata*[4,7] *and Hikaru Saito*[3,8,*]

[1] Department of Applied Science for Electronics and Materials, Kyushu University, Fukuoka 816-8580, Japan
[2] Department of Energy Science and Engineering, Kyushu University, Fukuoka 819-0395, Japan
[3] Institute for Materials Chemistry and Engineering, Kyushu University, Fukuoka 816-8580, Japan
[4] Department of Advanced Materials Science and Engineering, Kyushu University, Fukuoka 816-8580, Japan
[5] Department of Materials Science and Engineering, Virginia Tech, Blacksburg, VA 24061, USA
[6] Reactor Materials and Mechanical Design Group, Energy and Environmental Directorate, Pacific Northwest National Laboratory, WA 99352, USA
[7] The Ultramicroscopy Research Center, Kyushu University, Fukuoka 819-0395, Japan
[8] Pan-Omics Data-Driven Research Innovation Center, Kyushu University, Fukuoka 816-8580, Japan

*corresponding authors

A. Image distortion correction

B. Collection of training data for deep learning

C. Diffraction condition for imaging dislocations

D. Performance evaluation of U-Net-based and BM3D-based noise filters

E. Optimization of BM3D-based noise filter

F. Extraction of local intensity centers from tomography data

G. Image binarization

H. Tilt angle calibration for rapid tomography

A. Image distortion correction

Rapid scan in STEM brings about image distortion in the X direction because the X axis corresponds to the fast scan axis as shown in Fig. S1a. To calculate and correct the distortion, we firstly compare the rapidly scanned image of the Au grating sample (Fig. S1b) to relatively slowly scanned that (Fig. S1c). Note that the former in the figure is denoised by DCFI technique. The rest of this section describes how we corrected the distortion.

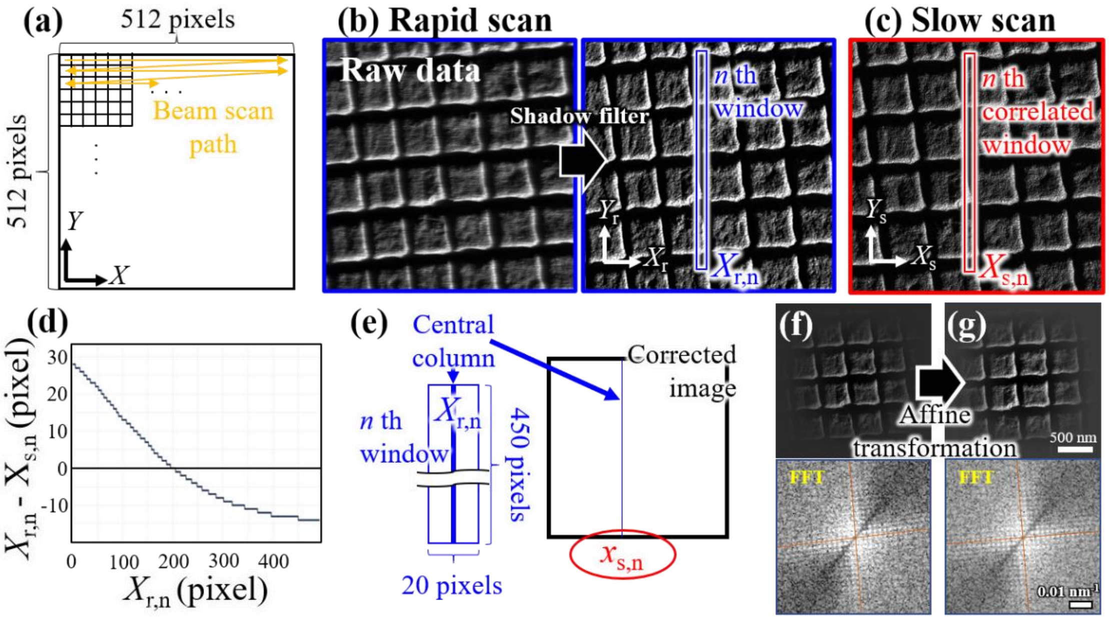

Figure S1 Distortion correction process. (a) Schematic drawing of scan process of STEM, and (b) nonlinearly distorted image due to rapid scan. To let the rapid scan image and (c) the slow scan image correspond to each other, a part of the rapid scan image, which is indicated as the n-th "window" at $X_{r,n}$, is to be compared with the n-th window at $X_{s,n}$ in the slow scan image. From the window pair which shows the highest cross correlation, we calculate the difference of position between the two, as shown in (d), enabling (e) the correction of the distortion. Linear distortion in the slow scan image is corrected by Affine transformation estimated from its FFT image ((f) and (g)).

First, a "shadow" filter is applied to the raw image of rapid scan to reduce image blur in the X direction, which is probably originating from a finite time width of the electron detector. This filter is a kind of differential filter, which combines the original image and its differential, expressed as

$$\begin{bmatrix} -1 & 0 & 1 \\ -2 & 1 & 2 \\ -1 & 0 & 1 \end{bmatrix}.$$

Then a partial image corresponding to the "slit-shaped window" depicted in Fig. S1b is extracted from the rapid scan image. The horizontal and vertical sizes of this window are 20 pixels and 450 pixels, respectively. The central axis of the window is placed at a horizontal position $X_{r,n}$. Then the corresponding partial image in the slow scan image is searched with cross-correlation coefficient. The central position of the found partial image is named as $X_{s,n}$ in Fig. S1c. Figure S1d shows measured difference of the horizontal position $X_{r,n} - X_{s,n}$, indicating monotonically decreasing. This result quantitatively shows how the rapid scan image is shrunken in the x direction comparing to the slow scan image. Importantly, the slope of the curve in Fig. S1d is not constant, meaning that the distortion correction should be done point by point. In Figs. 3 and 4 of the main text, the obtained coordinate transformation from $X_{r,n}$ to $X_{s,n}$ described in Fig. S1e was applied for all the rapid scan images.

Unfortunately, there still be linear image distortion even in the slow scan image. This linear image distortion can be recognized as non-orthogonal bright spot array in the fast-Fourier-transform (FFT) pattern of Fig. S1c (Fig. S1f), which should be orthogonal since this periodicity comes from the square lattice pattern formed in the standard sample. This linear image distortion can be corrected by a simple affine transformation which was optimized so that the FFT pattern becomes a square lattice (Fig. S1g). The image dimension was also calibrated by the spacing of the bright spot array in the FFT pattern, which corresponds to the square lattice pattern of 463 nm

period (nominal value). In Figs. 3 and 4 of the main text, scale bars determined by the above calibration were attached in all the rapid scan images after being corrected regarding the nonlinear distortion and the linear distortion.

Although the image distortion was expected to be limited to the *X* direction, we have calculated that in the *Y* direction to make sure that there is no affection in the *Y* direction. The same procedure in the case of the *X* direction was applied here but the dimensions of the window were 20 pixels and 300 pixels for the vertical and horizontal directions, respectively. As a result, we have confirmed that there was no detectable distortion in the *y* direction for everywhere.

B. Collection of training data for deep learning

Because the effective thickness, which an electron beam penetrates through, continuously changes as the specimen tilts, the signal-to-noise ratio also differs for each angle. Hence, training data collected from a single tilt angle was supposed to be insufficient for a noise filter applicable to images with a wide range of tilt angle. That is why we collected the training data from 5 tilt-angles (0°, 20°, 40°, 60° and 70°) as explained in the main text.

We confirmed that the training data and the rapid tilt-series images (Tilt-series 1) were almost comparable regarding the original intensity histogram. Figure S2 shows how we evaluated the intensity range of the training data and the rapid tilt-series images. In the figure, the histogram (Fig. S2b) was obtained from a reference (DCFI) image at 0° (Fig. S2a). P_1 and P_2 in Fig. S2b are defined as the lower and larger intensity, respectively, where the number of pixels is the half maximum. We measured P_1 and P_2 of the reference (DCFI) images for 5 different tilt angles for each of the areas as shown in Fig. S3, where the mean and the standard deviation for each of the

tilt angles are also shown. Comparing P_1 and P_2 of the training data and those of the rapid tilt-series images in Fig. S4, they are sufficiently close, and thus the used training data satisfied a requirement in terms of the intensity range. That might be one of the reasons why the deep learning successfully implemented in this study.

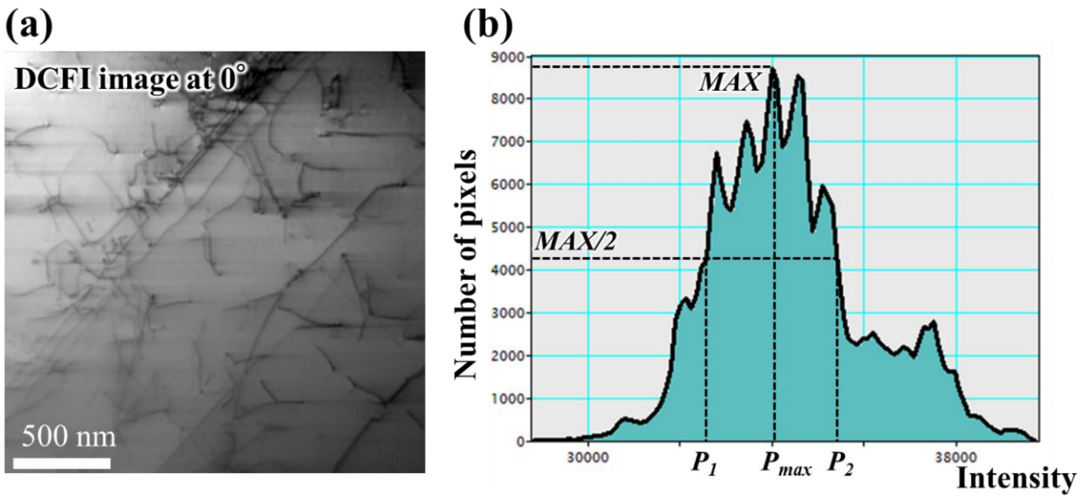

Figure S2. An example of how to obtain intensity range. (a) one of the DCFI images in training data acquired at 0° and (b) its contrast histogram. At P_1 and P_2, the number of pixels satisfies the half maximum (*MAX/2*). Note that the DCFI image contrast is optimized for visibility.

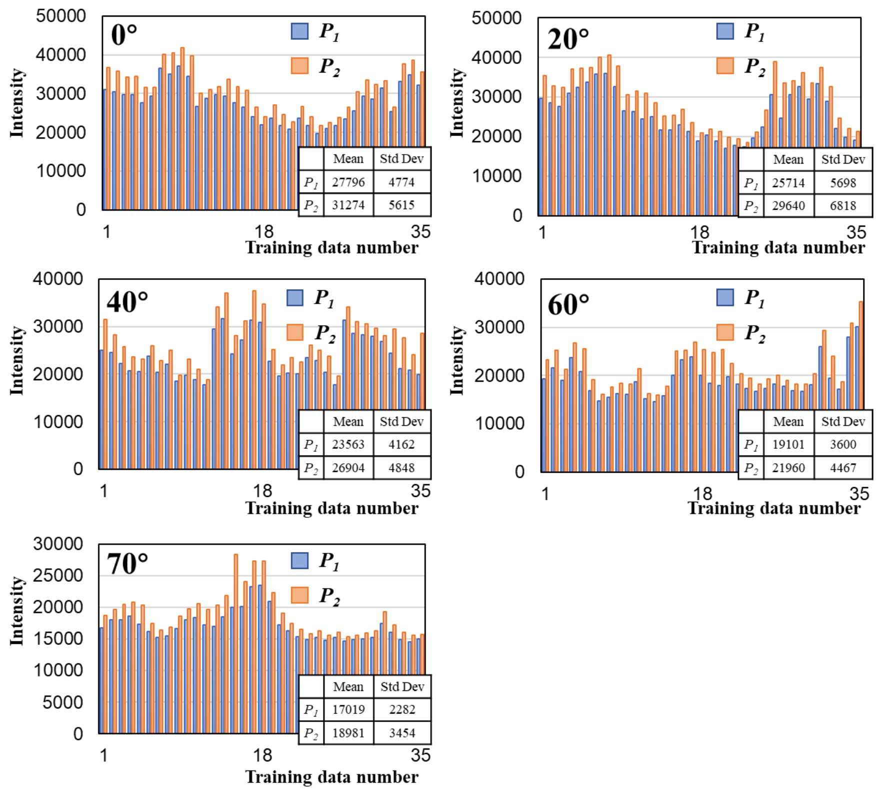

Figure S3. P_1 and P_2, which are defined in Fig.S2, of training data obtained at 35 areas for 0°, 20°, 40°, 60° and 70°. Mean and standard deviation of P_1 and P_2 are also shown.

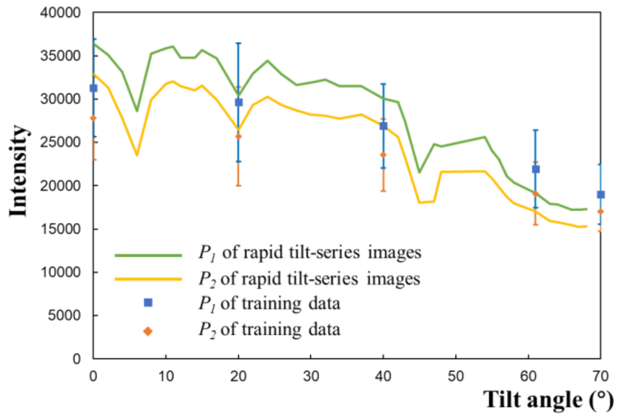

Figure S4. P_1 and P_2 as a function of the tilt angle measured from the reference images for the training process (dots) and the rapid tilt series images (solid lines). For the training data, the mean and standard deviation among the different areas were calculated and plotted as the dots and error bars.

C. Diffraction condition for imaging dislocations

All the dislocation images in this study were obtained under excitation of the 200 diffracted beam as shown in Fig. S5.

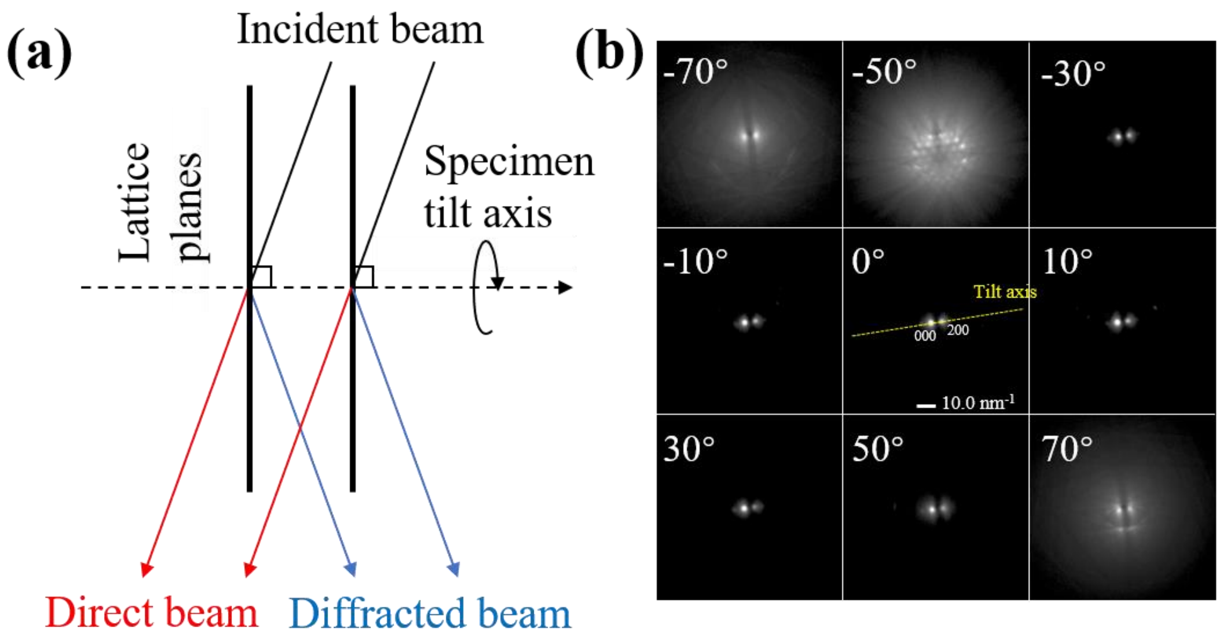

Figure S5. (a) Schematic drawing of the diffraction condition for the dislocation imaging. (b) Diffraction patterns at several selected tilt angles. Excitation of the 200 diffracted beam was maintained through all the tilt angles as shown in the diffraction patterns by adjusting the crystal orientation so that the $\mathbf{g}_{200}$ reciprocal lattice vector was parallel to the tilt axis.

D. Performance evaluation of U-Net-based and BM3D-based noise filters

The performance of the noise filters in this study was evaluated by the relative dislocation contrast and the relative width of the visualized dislocation line as shown in Tables 2 and 3 in the main text. The displayed values were calculated as ratios to the reference images (averaged images by 50 frames). Figure S6 shows examples of test data used for evaluation. Note that any images in the test data were not included in the training data.

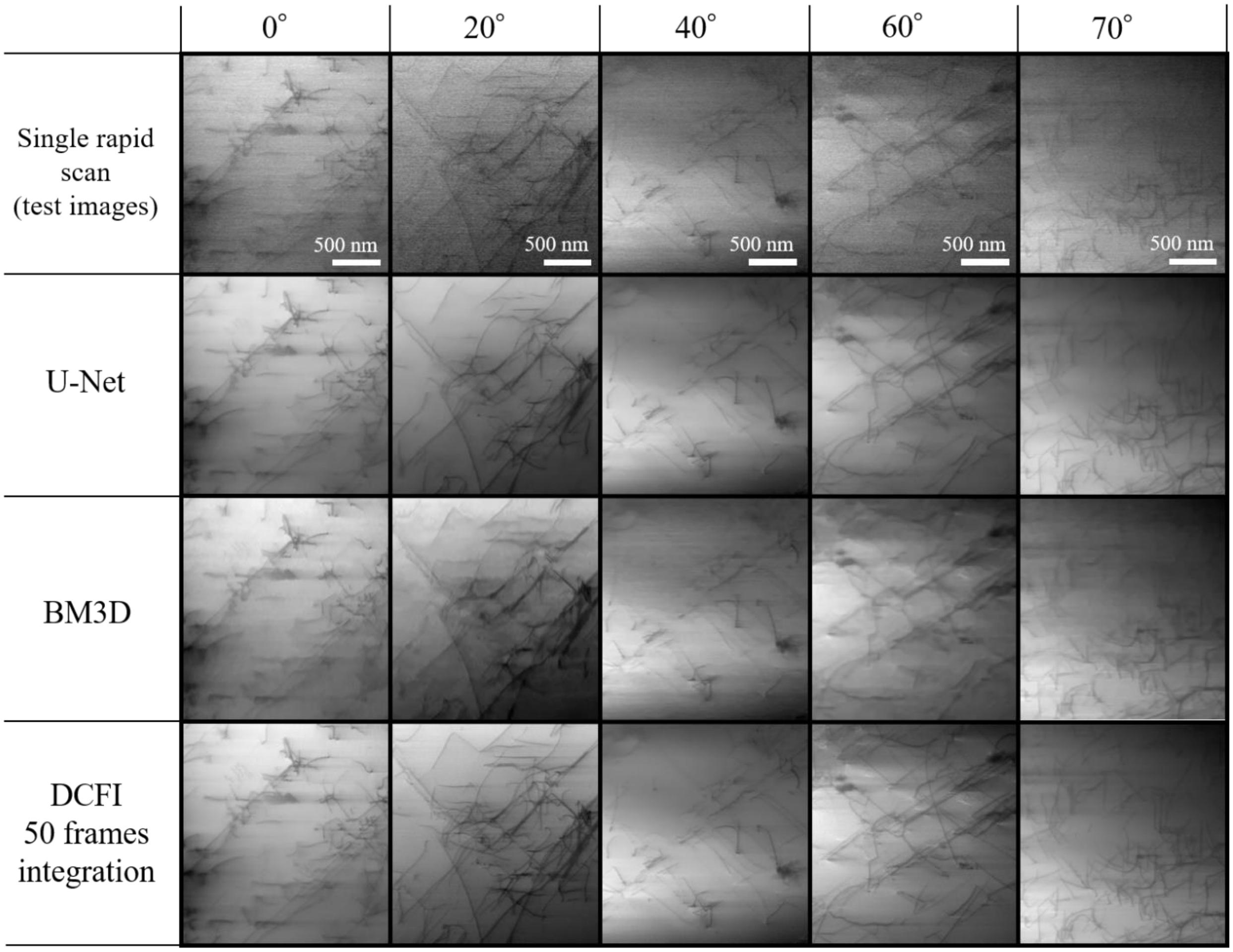

Figure S6. Examples of test data for 0°, 20°, 40°, 60° and 70° comparing to their denoised images by the U-Net and BM3D filters, and the reference images.

Figure S7 shows the distribution of pixel value extracted along a line crossing a dislocation in a reference image, where the line profiles obtained from the corresponding BM3D-based denoised image and the U-Net-based one are indicated by the green and red line, respectively. Here, the contrast of dislocation was defined as the depth of the local minimum from the surrounding intensity (indicated as “1” in Fig. S7). In the case of Fig. S7, the left side of the dislocation has higher intensity than the right side. For such an asymmetric dip, we always selected the higher side as the standard level for the contrast measurement. The width of contrast was measured at the level of the half depth of the red bar 1 (indicated as “2” in Fig. S7). We have obtained 10 line-profiles from the images for 0°, 20°, 40°, 60° and 70° (Fig. S6), respectively, and measured the contrast and the width (1 and 2) following the above procedure to evaluate deterioration of the spatial resolution and visibility.

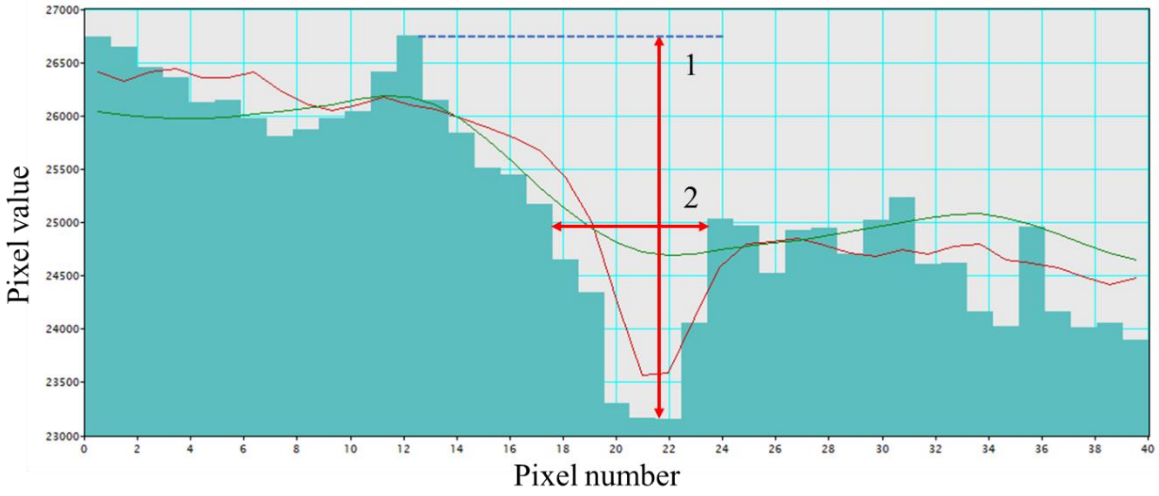

Figure S7. Distribution of pixel value extracted along a line crossing a dislocation. Line profiles at same position obtained from BM3D-based denoised image and U-Net-based one are indicated by green and red line, respectively.

E. Optimization of BM3D-based noise filter

To utilize BM3D-based denoising, we needed to choose noise of image and its variance in advance. Nine types of noise are assumed in the BM3D-based noise filter used in this study [S1], where we can choose the optimum noise type and adjust the variance of the noise amplitude distribution. We determined the best choice of the noise type and the noise variance by evaluating the PSNR relative to the reference image (averaged image). Let a noise called "gw" is as an example case, figure S8 shows a variation of PSNR, demonstrating that the peak (the best performance of BM3D-based denoising) appears around 0.002 of the assumed noise variance. By calculating the PSNR over 0.0001~0.0125 of the noise variance, and over all the nine types of noise, we determined the best combination of those for each image. Such optimized noise filter was to be compared to U-Net-based noise filter.

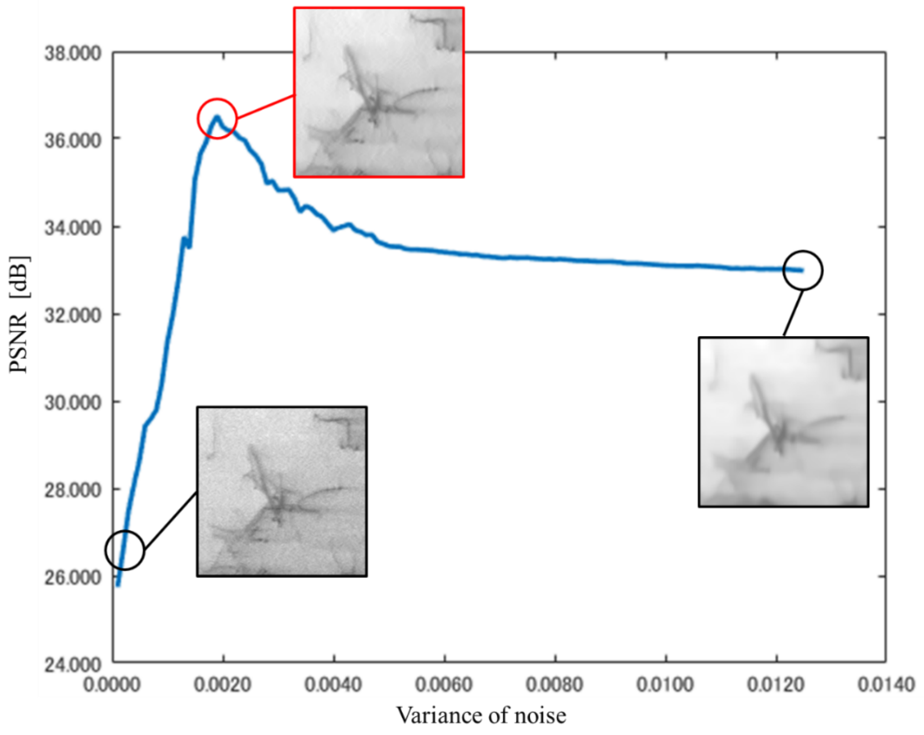

Figure S8. Variation of PSNR with standard deviation of noise.

F. Extraction of local intensity centers from tomography data

Figure S9 shows a representative *yz* cross-section of the noise filtered Rapid 3D, where the seven cross-sections of dislocations form local maxima (indicated by red ellipses). In order to determine a reasonable area for summation of the weighted average (Eq. 1 in the main text), we first detected the local maxima for each of the dislocations as shown in the Fig. S9. Each of the summation was performed within the 100 nm × 100 nm area centered at the found local maximum.

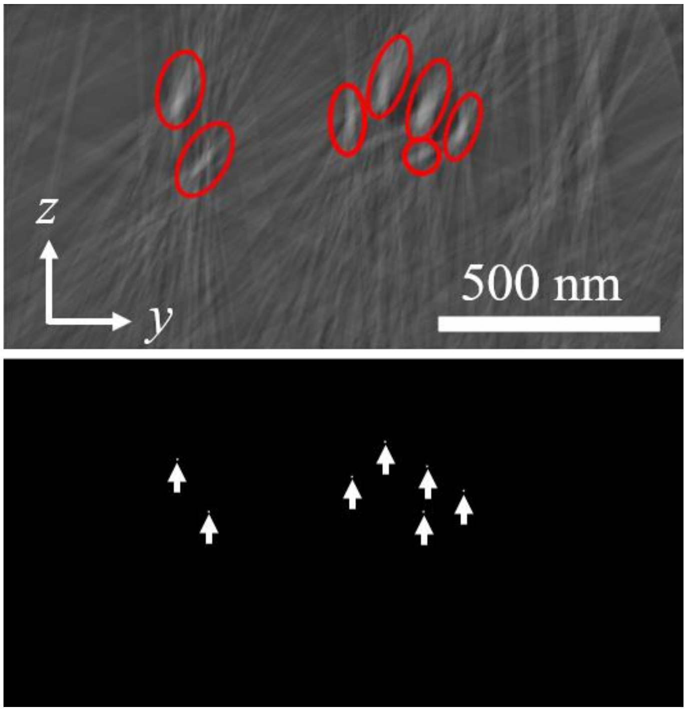

Figure S9. Representative *yz* cross-section of the noise filtered Rapid 3D (top panel) and the found local maxima (arrowed dots in the bottom panel).

G. Image binarization

For ideal tomographic reconstruction, tilt-series images must satisfy the projection requirement, that is, intensity in each of the tilt-series images must be a monotonic function of the targeted physical quantity integrated along the electron beam trajectory. However, raw tilt-series images of dislocations in a slab-shaped sample hardly satisfy the projection requirement, since the dislocation contrast depends not only the thickness of lattice strain distribution but also the total sample thickness (thickness of the surrounding matrix).

To eliminate such an undesirable sample-thickness dependence, the following binarization was performed on each of the images in both Tilt-series 2 (rapid STEM tomography) and Tilt-series 3 (slow STEM tomography) before 3D reconstruction, by using ImageJ and OpenCV available in Python. First, we subtracted a background obtained by lowpass filter from a targeted image. The lowpass filter in this study extracted lower frequency component within the circle with radius of 6 pixels from the center of fast Fourier transform (FFT) image of the original image. Then the subtracted image contrast was inverted (hereafter dislocation lines become white), followed by global thresholding of the image for eliminating the residual intensity in the flat background area, where the intensity less than 2 % of the maximum intensity was substituted by 0. The processed image was binarized through the Gaussian adaptive binarization where a threshold $T(x, y)$ at a position (x, y) is calculated by the Gaussian-weighted sum as follows,

$$T(x,y) = \sum_{i=1}^{i\times i} W_i \times L_i, \quad \text{(G.1)}$$

which is calculated within a $i \times i$ square block centered at the position (x, y). i must be an odd number. W_i is the gaussian-weighted of each pixel in the calculation block, and $\sum_{i=1}^{i\times i}(W_i) = 1$, L_i is intensity of each pixel in the block. We determined the block size i as 7, resulting in the comparable width of the dislocation contrast as that in the original images. This binarization

process almost successfully highlighted dislocations. However, some small sized debris was left in the flat background areas because of tiny fluctuations of background pixel value. Therefore, objects with area of less than 50 pixels were removed.

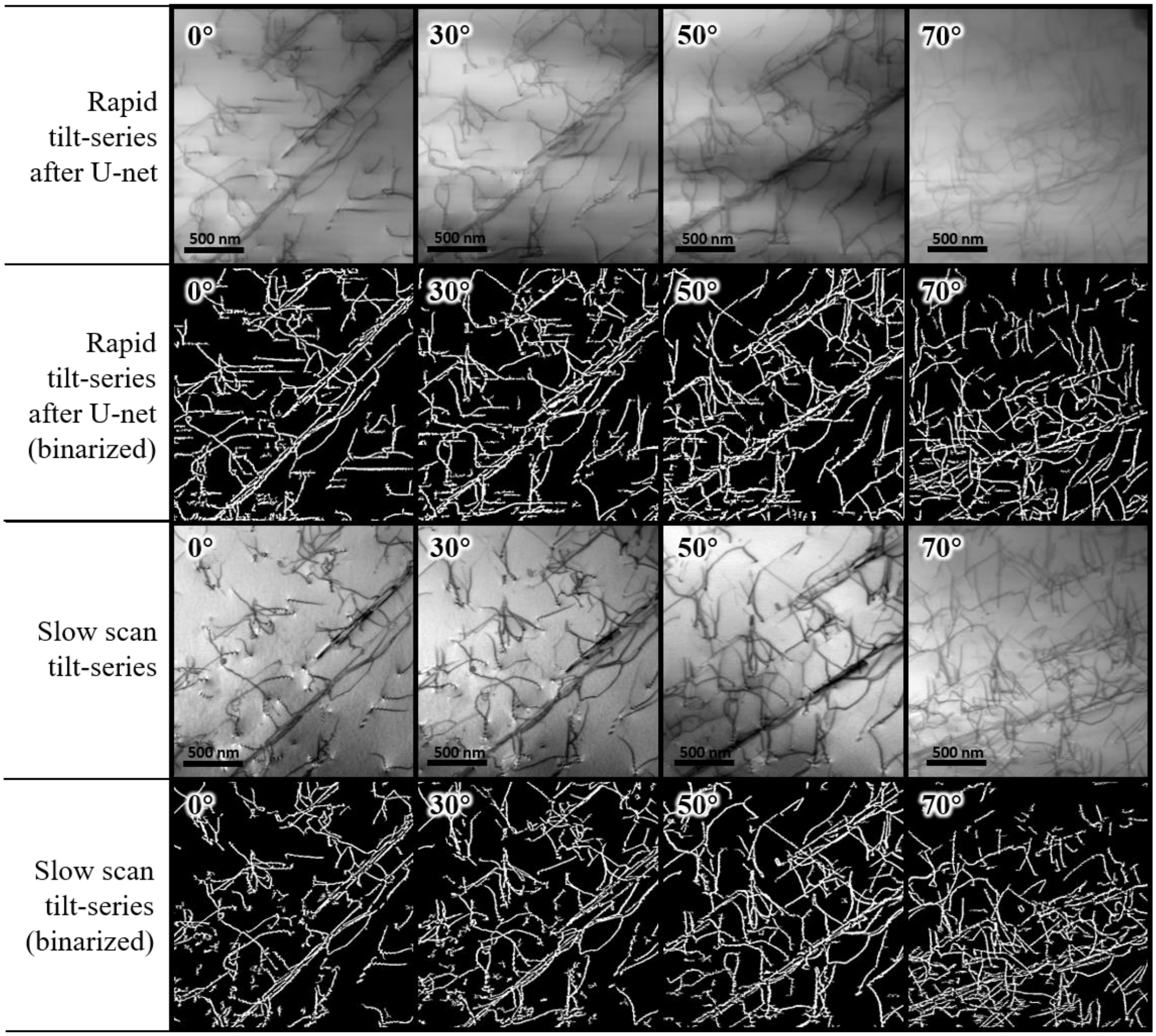

Figure S10. Several images selected from Tilt-series 2 and 3, and their binarized images.

H. Tilt angle calibration for rapid tomography

Unlike a conventional STEM tomography, in the rapid STEM tomography, it is hard to record the accurate tilt angle from the angle indicator of the goniometer because the sample tilts at non-uniformly high speed without stopping from the initial angle to the final one. Therefore, we calculated the tilt angle for each of the frames from the tilt-series images themselves.

At first, we found a pair of dislocation tips as "feature points" in an image, which are to be also marked throughout the series of images (Fig. S11a). If the line connecting the feature points is not tilted, the vertical distance d between the feature points should be longest as shown in Fig. S11b. Therefore, the tilt angle α^a in a-th frame can be estimated by comparing the distance d^a throughout the entire frames, i.e., it is expressed as

$$\alpha^a = \cos^{-1}(d^a/d_{max}) \quad \text{(H.1)},$$

where d_{max} is the maximum distance in a tilt series. When $d^a = d_{max}$, α^a becomes 0°, meaning that the line connecting the feature points horizontally lies in the sample. Since the 3D reconstruction just requires relative angles among the frames, we can arbitrarily define the origin of the tilt angle. In other words, the surface of the slab sample is not needed to be perpendicular to the electron beam at 0° of the tilt angle. For the purpose of error reduction, we measured α_b^a (b=1,2, ⋯,10) from 10 pairs of feature points. However, we cannot simply average α_b^a among the different pairs since the connecting lines are tilted from each other by certain angles. Such an angle difference of the b-th pair to the 1st pair is calculated as

$$\frac{1}{n}\sum_{a=1}^{n}(\alpha_b^a - \alpha_1^a) \quad \text{(H.2)},$$

where n is the number of total frames. By subtracting this angle difference from the original tilt angle, i.e.,

$$\alpha_b^a \to \left\{\alpha_b^a - \frac{1}{n}\sum_{a=1}^{n}(\alpha_b^a - \alpha_1^a)\right\} \quad \text{(H.3)},$$

all the angle origins are unified to the origin of α_1^a. Finally, the tilt angle of a-th frame is estimated as,

$$\alpha^a = \frac{1}{m}\sum_{b=1}^{m} \alpha_b^a \qquad \text{(H.4)},$$

where m is the number of calculated distances d^a (m=10 in this study). In this way, we obtained a relationship between the frame number and tilt angle as shown in Fig. S12.

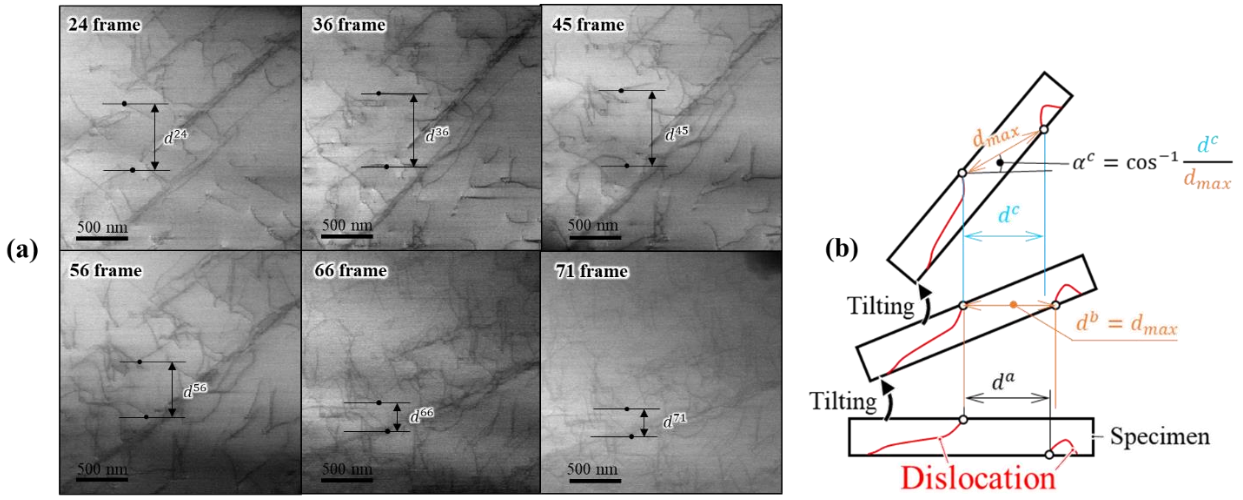

Figure S11. An example of how to obtain the vertical distance between two points, namely "feature points". (a) Representative images in the rapid tilt series with markers indicating the vertical distance between the feature points. Since these images were obtained as projection of a specimen, the distance changes as it tilts, as schematically drawn in (b).

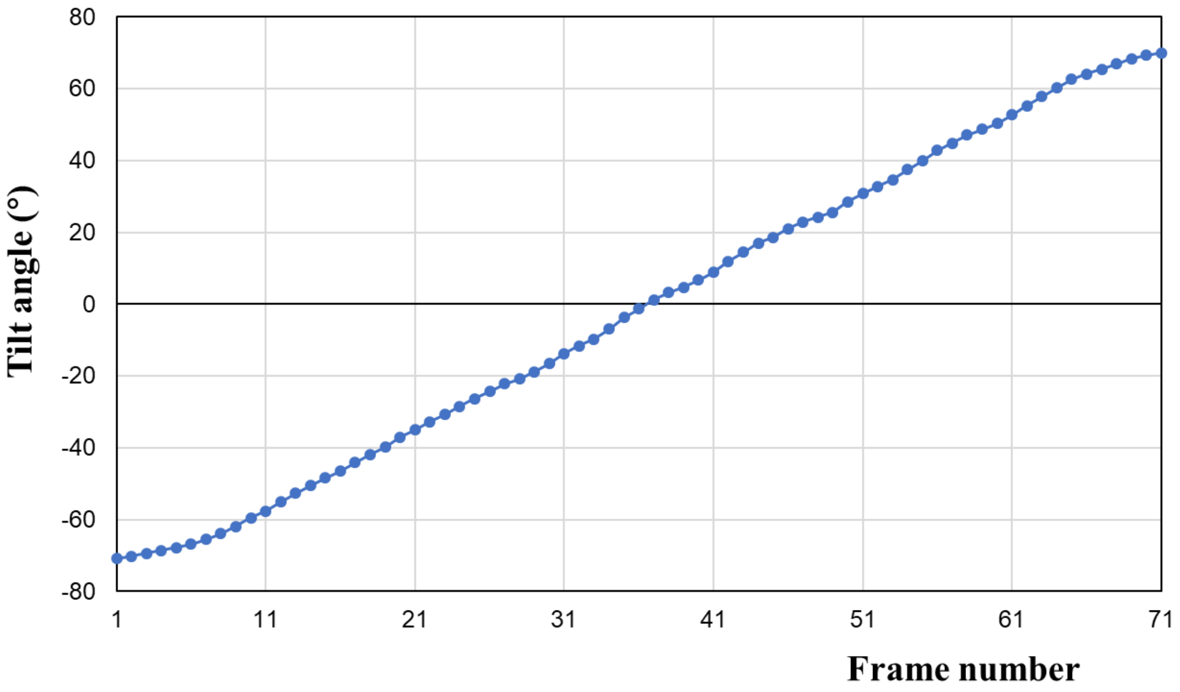

Figure S12. Variation of tilt angle with frame number.

References

[S1] Y. Makinen, L. Azzari, and A. Foi, "Collaborative filtering of correlated noise: Exact transform-domain variance for improved shrinkage and patch matching", IEEE Trans. Image Process., 29 (2020), pp. 8339–8354.

Supplementary video

Supplementary video S1. Raw tilt-series images (Tilt-series 1).

The attached scale bar includes 10 % errors at most because these tilt-series images are before the distortion correction.

Supplementary video S2. Tilt-series images processed by the U-Net-based noise filter (Tilt-series 2 before the distortion correction).

The attached scale bar includes 10 % errors at most because these tilt-series images are before the distortion correction.

Supplementary video S3. Parallel view of the 3D reconstructed data from Tilt-series 2 (Rapid 3D). The reconstructed 3D volume of 1797 × 1792 × 912 nm.

Supplementary video S4. Perspective view of the 3D reconstructed data from Tilt-series 2 (Rapid 3D). The reconstructed 3D volume of 1797 × 1792 × 912 nm.

Supplementary video S5. Parallel view of the 3D reconstructed data from Tilt-series 3 (Slow 3D). The reconstructed 3D volume of 1797 × 1792 × 912 nm.

Supplementary video S6. Perspective view of the 3D reconstructed data from Tilt-series 2 (Slow 3D). The reconstructed 3D volume of 1797 × 1792 × 912 nm.

Supplementary video S7. Parallel view of superposition of denoised Rapid 3D and Slow 3D. The reconstructed 3D volume of 1797 × 1792 × 912 nm.

Supplementary video S8. Perspective view of superposition of denoised Rapid 3D and Slow 3D. The reconstructed 3D volume of 1797 × 1792 × 912 nm.
